# Editorial: Break the mental health stigma: loneliness

**DOI:** 10.3389/fpsyt.2024.1373524

**Published:** 2024-02-01

**Authors:** Natalia Martín-María

**Affiliations:** ^1^ Department of Biological and Health Psychology, Universidad Autónoma de Madrid, Madrid, Spain; ^2^ Centro de Investigación Biomédica en Red de Salud Mental, CIBERSAM, Madrid, Spain

**Keywords:** cognition, depressive symptoms, rural communities, randomised control trial, humanoid robot intervention

The Frontiers Research Topic entitled: “*Break the Mental Health Stigma: Loneliness*” is aimed to provide insights into research and interventions tackling loneliness so that these were translated into clinical applications and public health policies.

Loneliness is a negative feeling defined as the discrepancy between one’s desired and one’s actual grade of social relationships (1). A recent systematic review that analysed more than one hundred countries around the world revealed that a considerable segment of the population experienced loneliness (2). It could jeopardise health and wellbeing, so it has been identified as a significant public health issue (3). Research in loneliness is relatively new but enormous in number of publications. Despite everything, there are still many areas to explore. It is relevant to understand the impact of loneliness in people from rural communities, places where studies have paid less attention. Furthermore, it is still important to study whether the COVID-19 pandemic has affected loneliness and social isolation, especially in vulnerable groups such as older people.

Four articles were included in this Research Topic collection, two of them were reviews, one comprises a cross-sectional study, and the last one was a study protocol for a pilot randomized control trial.

The scoping review of Kassam and McMillan aimed to examine the impact of social isolation and loneliness on cognition in older adults, analysing quantitative and longitudinal studies that measured both variables at two times points in the course of COVID-19 pandemic. CINAHL, Medline, PubMed, and PsycINFO databases were used. In six out of seven studies incorporated in the conclusive data analyses, loneliness and social isolation were related to worse cognitive performance. Hussain et al. carried out a quantitative and qualitative systematic review, focused on analyse loneliness and social networks in older adults of rural communities, who represent a fifth of the total population of older adults. The following databases were consulted: MEDLINE (Ovid), CINAHL (EBSCO), Embase (Ovid), PsycINFO, Scopus, SocINDEX (EBSCO). They found that social networks of older adults living in rural areas comprise family, friends, and neighbours. These relationships evolved by joining in physical, social, and religious activities. After reviewing interventions to tackle loneliness and social isolation, they conclude that the establishment of social bonds and consistent engagement with others were crucial elements in such purpose.

Homosexuality in Chinese men and its connection to loneliness and depressive symptomatology was the selected topic of the cross-sectional study of Liu et al. A total of 655 men aged 15 or older were under study. The absence of social support and low self-esteem were identified as risk factors for experiencing loneliness and depressive manifestations. Particularly, among men who have sex with men, those who were in their youth, unmarried, and presented lower levels of self-esteem exhibited heightened vulnerability to the effects of depressive symptoms on loneliness.

Finally, the article of Lavin et al., showed a protocol for a randomized controlled trial to contrast the results of a humanoid robot intervention to treatment as usual on loneliness and mental health variables (depression, stress, anxiety, quality of life, and a decrease in the use of urgent healthcare services) in long-term care residents. The robot is called Grace and it was first developed to interact with older people and those alone by the COVID-19 pandemic. They also pretend to use qualitative methodology to measure viability and adequacy of the study among participants.

In conclusion, this Research Topic spotlight the need to continue investigating into loneliness in order to curb it, especially among older population and other vulnerable groups. As can be gathered from the studies collected above, more intervention studies focused on prevent and deal with loneliness still are needed.

## Author contributions

NM-M: Writing – original draft, Writing – review & editing.
